# A giant peritoneal simple mesothelial cyst: a case report

**DOI:** 10.1186/1752-1947-5-361

**Published:** 2011-08-10

**Authors:** Abdelmalek Ousadden, Hicham Elbouhaddouti, Karim Hassani Ibnmajdoub, Taoufiq Harmouch, Khalid Mazaz, Khalid AitTaleb

**Affiliations:** 1 Service de Chirurgie Viscérale, Hôpital des Spécialités, CHU Hassan II, Route de Sidi Harazem, Fès, 30070, Morocco; 2 Laboratoire d'anatomie pathologique, Hôpital des Spécialités, CHU Hassan II, Route de Sidi Harazem, Fès, 30070, Morocco

## Abstract

**Introduction:**

A peritoneal simple mesothelial cyst is a very rare mesenteric cyst of mesothelial origin. The size of this lesion usually ranges between a few centimeters and 10 cm. It is usually asymptomatic, but occasionally presents with various, non-specific symptoms, which makes correct pre-operative diagnosis difficult. We present a case of a giant peritoneal simple mesothelial cyst that was successfully managed by complete surgical excision which is the treatment of choice.

**Case presentation:**

A 21-year-old Caucasian Moroccan woman with vague abdominal discomfort and associated distention, during the previous 2 years, without other symptoms, presented to our hospital. Her past medical history was unremarkable. On physical examination, a mobile, painless and relatively hard abdominal mass was palpated. The laboratory examination and abdominal radiograph were unremarkable. Abdominal radiologic imaging showed a cystic mass of 35 × 20 × 10 cm that occupied the entire anterior and right abdominal cavity. Radical excision of the cyst was performed by midline laparotomy without any damage to the adjacent abdominal organs. The histopathological diagnosis was simple mesothelial cyst. The postoperative course was uneventful with no recurrence.

**Conclusion:**

**A **peritoneal simple mesothelial cyst is a quite rare abdominal tumor, that must always be considered in differential diagnosis of pelvic cystic lesions and other mesenteric cysts. The treatment of choice is the complete surgical excision of the cyst.

## Introduction

According to Perrot classification, the peritoneal simple mesothelial cyst (PSMC), benign cystic mesothelioma and malignant cystic mesothelioma are mesenteric cysts (MC) of mesothelial origin [1]. The other MC types are non-pancreatic pseudocysts, dermoid cysts and cysts of lymphatic, enteric or urogenital origin [1]. PSMC is very rare, with only about 900 reported MC cases in the literature [2,3]. The cyst size ranges from a few centimeters to 40 cm [2,4,5]. The PSMC is usually asymptomatic, but occasionally presents with various, non-specific symptoms. The lack of specific symptoms and the rarity of PSMC, makes correct pre-operative diagnosis difficult.

We present the case of a woman with a giant PSMC that was successfully managed by complete surgical excision, which is the treatment of choice of this lesion.

## Case presentation

**Figure 1:**
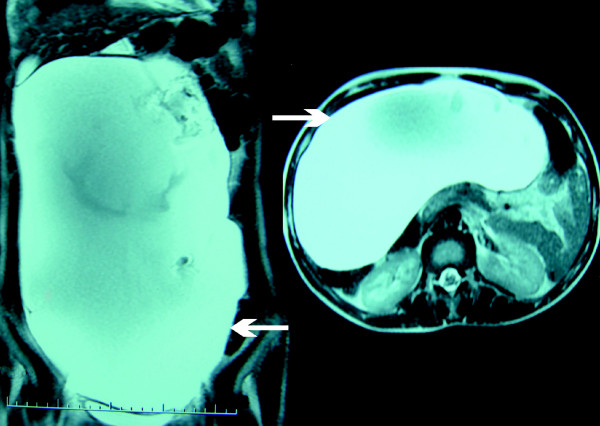
**Abdominal MRI revealing a giant peritoneal cystic tumor**.

A 21-year-old Caucasian Moroccan woman with vague abdominal discomfort and associated distention, during the previous two years, without other symptoms was admitted to our hospital. Her past medical history was unremarkable. On physical examination a mobile, painless and relatively hard abdominal mass was palpated. The laboratory examinations were unremarkable and tumor markers were normal. An abdominal radiograph showed a normal intestinal gas pattern. Abdominal ultrasound examination showed an anechoic cystic mass filling the entire anterior and right abdominal cavity. There was no pathological intestinal segment or intra-peritoneal free or loculated fluid. Abdominal computed tomography and magnetic resonance imaging showed a giant mass of 35 × 20 × 10 cm in the abdominopelvic cavity that had no association with other abdominal organs (Figure 1).

**Figure 2:**
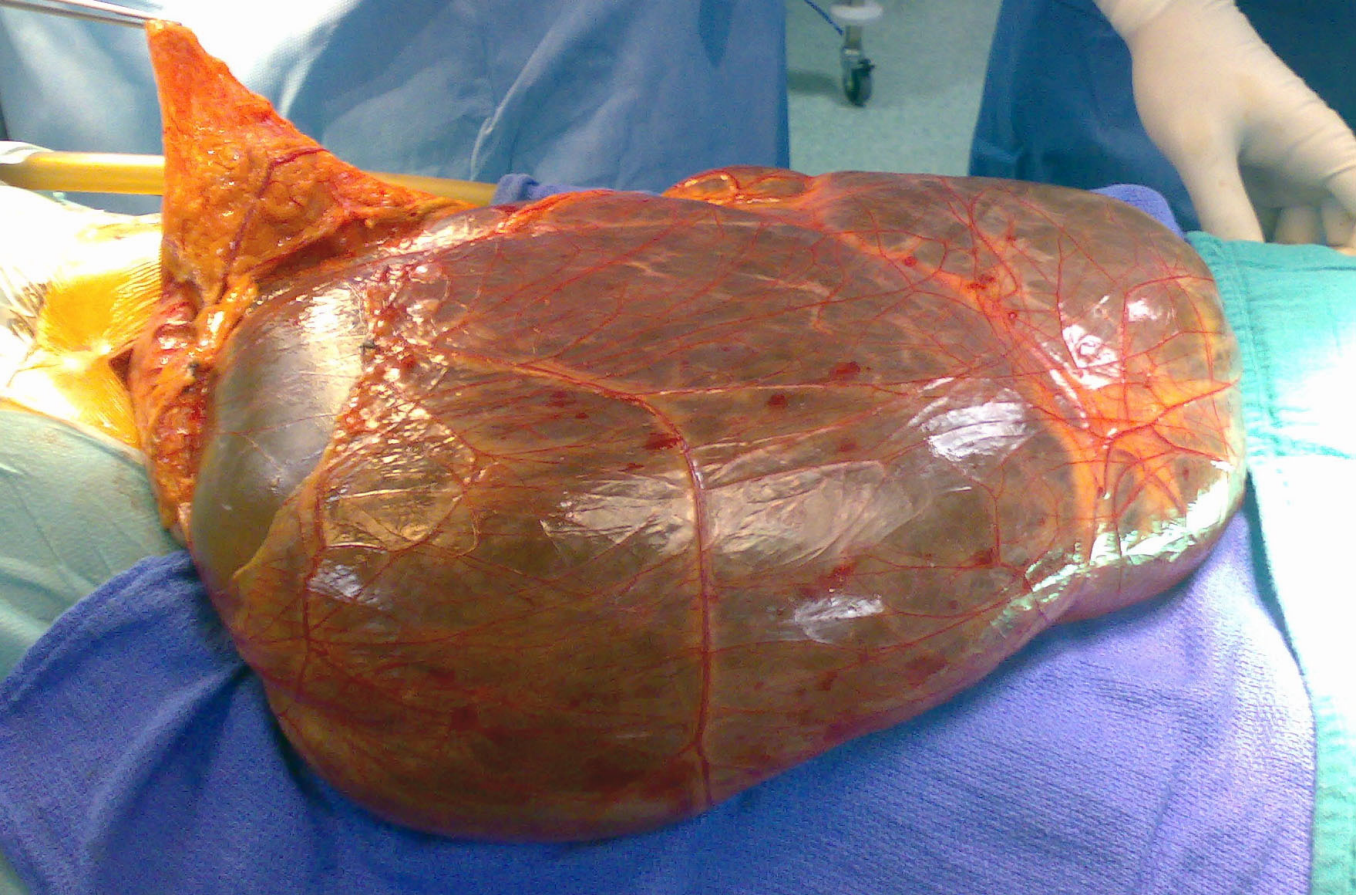
**The extracted tumor, at laparotomy, is a giant cyst with a thin wall and serous fluid content**.

**Figure 3:**
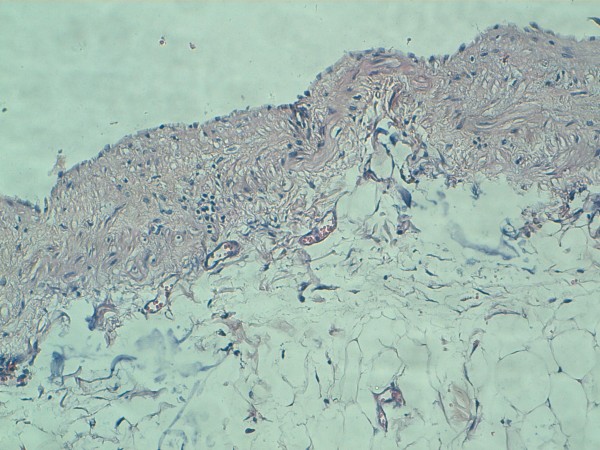
**The cyst wall is fibrous, lined by regular mesothelial cells showing no atypia and no mitosis**. (Hematoxylin and eosin, X20).

Midline laparotomy revealed a giant abdominopelvic cyst associated with the transverse mesocolon (Figure 2). Radical excision of the cyst was performed without any damage to the adjacent abdominal organs. Macroscopically the mass was unilocular and contained approximately 5000 ml of serous fluid. The histopathological diagnosis was simple mesothelial cyst having a fibrous wall and lined by regular mesothelial cells showing no atypia and no mitosis (Figure 3). The post-operative course was uneventful. All parameters of the patient were normal and she was discharged on the following day. Six months after surgery she remains completely asymptomatic with no recurrence.

## Discussion

PSMC is most likely the result of the congenital incomplete fusion of the mesothelial-lined peritoneal surfaces. Therefore, PSMC is located in the small bowel, the mesentery, the mesocolon and the omentum [6,7]. PSMC occurs in children and young adults and usually does not occur in older people [6]. Pathological examination reveals that PSMC is a thin-walled, unilocular cyst that usually contains serous material [7]. The inner surface of PSMCs is lined by flat, cuboidal or columnar mesothelial cells and its wall are fibrotic without any lymphatic or musculous structures [2,7]. The cytology of PSMC shows rounded cells with a regular round nuclei, a prominent single nucleoli and abundant cytoplasm [2]. An immunohistological analysis can achieve further characterization of mesothelial cells which are negative for Factor VIII and CD31 and positive for total keratin, vimentin, and ethidium monoazide [2].

Correct preoperative diagnosis is usually based on clinical examination and radiographic imaging. It is a quite difficult diagnosis due to the rarity of this lesion and the lack of specific clinical presentation, which depends on size, and is asymptomatic [2,8]. When PSMC increases in size, common symptoms, due to the compressive effect of the cyst on surrounding structures, such as abdominal pain, distension, bloating, constipation and vomiting can arise [2,8,9]. Clinical examination may find a painless compressible soft abdominal mass relatively mobile transversely [2,9]. The cyst may be giant, simulating ascites or an ovarian tumor. Acute abdomen due to complications including rupture, obstruction, inflammation, infection, torsion or hemorrhage within the cyst or, more rarely, ascitis, may also be present [2,3,9]. Variable, unspecific and indolent symptoms are more frequent in adults while acute abdomen is a typical clinical presentation in children [2].

Plain radiographs and barium studies are often normal or non-specific revealing a non-calcified mass that displaces the bowel [6,9]. Abdominal ultrasonography (US), computed tomography (CT) scan and magnetic resonance imaging (MRI) are more useful [7,10]. They can demonstrate the cystic character of the lesion, and determine size, location, relation to surrounding structures and features of the cyst's wall and contents [2,6]. In cases of PSMC, abdominal US demonstrates an anechoic mass with acoustic enhancement [6,7,9]. CT and MRI reveal a fluid-filled mass with low signal intensity on Ti-weighted images, no discernible wall and no internal septations [7,9].

The laboratory investigation does not usually yield any significant information. In addition, it is rarely necessary to perform additional diagnostic procedures that may further characterize the cyst, such as fine needle aspiration with cytological analysis or explorative laparoscopy [2].

The treatment of choice is complete surgical excision of the cyst by enucleation from surrounding leaves of mesentery [2,8-10]. This is usually easily feasible either by laparotomy or laparoscopy in appropriately selected patients [3,8-10]. The cyst size, its location and the level of the surgeon's experience may also influence the decision regarding the surgical approach [10]. In our case laparoscopic surgery was not possible due to the size of the cyst. To exclude malignant alteration and prevent complications, resection of adjacent organs may occasionally be necessary [2,3,8]. Cyst puncture, simple drainage and marsupialization are treatment options that should not be performed due to their low efficacy and high risk of complications [3,8,9].

A PSMC is benign and has a very favorable prognosis [9]. Its total excision is curative with minimal surgical complications, mortality and no risk of recurrence [2,3,9].

## Conclusion

Although PSMC is a quite rare abdominal tumor, it must always be considered in the differential diagnosis of pelvic cystic lesions and other mesenteric cysts. The treatment of choice is the complete surgical excision of the cyst.

## Consent

Written informed consent was obtained from the patient for publication of this case report and any accompanying images. A copy of the written consent is available for review by the Editor-in-Chief of this journal.

## Competing interests

The authors declare that they have no competing interests.

## Authors' contributions

AO, KA and HE operated on the patient. KHI took the photos. KM participated in the follow up. TH made the histopathological diagnosis. All authors participated in writing the case report and revising the draft. All authors read and approved the final manuscript.
